# Modeling Financial Insolvency and Income Loss Insurance in Head and Neck Cancer

**DOI:** 10.36469/001c.155597

**Published:** 2026-03-03

**Authors:** Ana V. Araujo, Murray J. Bartho, Garren M. I. Low, Ryan J. Li

**Affiliations:** 1 Oregon Health & Science University, Portland, Oregon; 2 Allegheny Health Network, Pittsburgh, Pennsylvania

**Keywords:** catastrophic insurance, financial insolvency, modeling, head and neck

## Abstract

**Background:**

Head and neck cancer (HNC) carries high morbidity, and its treatment can be functionally devastating, impacting a patient’s ability to work. While most patients have medical insurance benefits, studies on the impact of HNC on overall household finances have been limited.

**Objectives:**

This study explored the effect of HNC treatment on household finances and the feasibility of catastrophic income loss insurance.

**Methods:**

This cross-sectional study was based on a population-level survey of American adults. Participants, aged 35 to 64 years, were respondents to the US Federal Reserve 2023 Survey on Household Economics and Decisionmaking (SHED).

**Results:**

With total income loss, 16% of simulated HNC patients were insolvent after 3 months, rising to 49% at 6 months. With a 50% loss in income, 3% of patients were insolvent at 3 months, increasing to 5% at 6 months. If savings were liquid, 0.5% of patients were insolvent at 3 months, rising to 1.3% at 6 months.

**Discussion:**

Our findings underscore the substantial financial vulnerability faced by patients undergoing treatment for HNC. Even in a simulated model based on national economic data, nearly half of patients experiencing total income loss were insolvent by 6 months. Given the intensive and prolonged nature of HNC treatment, these financial challenges may compound physical and psychosocial stressors, affecting overall recovery and quality of life.

**Conclusion:**

The model suggested a need for more substantial income loss protection programs. Financial hardship applies to other cancer types and merits further study into the household financial impact of HNC and other cancers.

## INTRODUCTION

Head and neck cancers (HNC) are relatively rare diagnoses, comprising approximately 4% of all cancers annually within the US, or approximately 71 000 new cases per year.^1^ The treatment for HNC can be functionally devastating, frequently requiring one or more of surgery, radiotherapy, and systemic therapy. A common treatment pathway for advanced cancers involves surgical excision and reconstruction. If the patient has indications for adjuvant therapy, this is ideally started within 6 to 8 weeks of surgery. The decision for adjuvant therapy is guided by the surgical pathology report, imaging studies, and clinical judgment of the oncology team. Radiotherapy treatment has a duration of approximately 6 weeks. In the absence of complications, there is at minimum 3 months from treatment start to end dates. Treatment can frequently be delayed due to wound healing or medical complications, prolonging overall treatment time. Treatment modalities for HNC can be functionally devastating, affecting speech, voice, swallowing, facial movement, shoulder and neck movement, vision, and hearing. At the completion of treatment, many patients are temporarily or permanently physically and/or emotionally disabled for prolonged periods of time, typically 3 to 12 months, with substantial work time lost.^2–4^ Prior data reported up to 12% of patients who had been working prior to cancer treatment had not returned to the work force 15 months after treatment completion.^4^ Fortunately, around 95% of patients will have health insurance.^5^ Benefits typically cover a significant proportion of healthcare costs, if not all. Less clear is the change in economic reality that occurs for patients and their households due to lost income while they are undergoing treatment and recovery. Prolonged loss of income can occur both during and after HNC treatment. The proportion of patients unable to work for the first 6 to 12 months after treatment remains unclear. We analyzed the 2023 Federal Reserve Board Survey on Household Economics and Decisionmaking (SHED) database, which reports annually on the financial stability and economic welfare of American households. This study aimed to model (1) the effect of income loss during and after cancer treatment on the financial stability of American households and (2) actuarially fair catastrophic income loss insurance premiums.

## METHODS

The Institutional Review Board of Oregon Health & Science University exempted this retrospective study from review and waived informed consent. In this study, we used data from respondents of the US Federal Reserve 2023 population-based SHED. The SHED is performed annually to examine American household financial welfare. The 2023 survey considers the increased rates of savings during the COVID-19 pandemic.^6^ This survey provides household annual income and savings data and select expense data including housing (rent/mortgage), student loans, and medical expenses. For the purposes of our study, financial data used included household annual income and savings, and housing expenses only, given other expense data was largely incomplete. Statistical analyses were performed using RStudio v4.3.1 (R Foundation for Statistical Computing). The following packages were used in the actuarial and analytic processes of this study: ggplot2, dplyr, summary tools, writexl, gridExtra.

### Financial Stress Test and Financial Stability Model

We performed a series of financial stress tests to model the effect of income loss during and after cancer treatment on the financial stability of US households. To conduct the financial stress test, respondents were simulated to have a new diagnosis of HNC and planned to undergo treatment with a 3-month minimum time off work. For this model’s purposes, monthly expenses continued at the same rate and all respondents had health insurance. Respondents less than 35 years of age were excluded, as it is rare to develop HNC younger than this age. Three financial stress tests were applied to each household. The first test modeled no income for 3 months. The second test modeled a 50% reduction in income for 3 months, simulating lost work with some income support. The third test modeled complete income loss for 3 months but with the ability to liquidate retirement savings. Federal income taxes were calculated using progressive 2023 federal income tax brackets, and effective state income tax rates were set to 3.8% for all respondents given high variation across states.

### Catastrophic Income Insurance Model

We modeled the total US expenditure on annual premiums for a catastrophic income loss insurance program that would protect the entire income of policyholders for 1 year. Annual income data was extracted from the SHED 2023 database. Respondents aged 35 to 64 years, a group at risk for HNC and typically participating in the work force, were divided into three 10-year groups: 35 to 44, 45 to 54, and 55 to 64 years. According to the Social Security Administration, 1 in 4 (25%) 20-year-olds will suffer full disability (≥1 year without income) by age 65.^7^ We used actuarial disability probability tables published by the Social Security Administration, which accounted for gender differences and provided an annual disability probability for ages 35 to 64, to create the probability that a subject would be disabled at any given age.^8^

To estimate actuarially fair disability premiums for each household in the SHED ages 35 to 64, each household’s expected wealth was calculated based upon their disability probability and income (**Table 1**). For the purposes of this study, we assumed full population participation in a disability insurance program wherein all households preferred wealth certainty and were willing to pay an insurance premium to guarantee that their wealth would not change in a disabled state, aside from having paid their disability insurance premium. In other words, respondents were risk-averse and willing to pay a higher insurance premium to guarantee that their wealth would not change whether they were able to work, or disabled. Finally, we summed the premiums from each household to calculate a population-aggregate annual premium. According to the US Census Bureau, there were approximately 126 651 558 US persons between ages 35 and 64 years in 2023.^9^ We used a multiple of 22 150 (126 651 558/5718 survey respondents) to scale the premiums summed from the 5718 SHED respondents to the total population.

**Table 1.** Calculation of the Income Protection Insurance Premium for 1 Respondent of the 2023 SHED

**Respondent No.**	**Case ID 35**
Age group	55-64 years
Annual after-tax income	$87 500
Wg (wealth in well state)	$87 500
Prg (probability of well state)	0.835
Wb (wealth in disabled state)	0
Wb_SSDI (wealth in disabled state with SSDI benefits)	$17 868
Prb (probability of disabled state)	0.165
Expected wealth without SSDI: (Wg x Prg) + (Wb x Prb)	$73 063
Expected wealth with SSDI: (Wg x Prg) + (Wb_SSDI x Prb)	$76 010
Expected premium without SSDI: Annual after-tax income - (Wg x Prg) + (Wb x Prb)	$14 437
Expected premium with SSDI: Annual after-tax income - (Wg x Prg) + (Wb_SSDI x Prb)	$11 490

Abbreviations: SHED, Survey on Household Economics and Decisionmaking; SSDI, Social Security Disability Income.

## RESULTS

**Figure 1.**
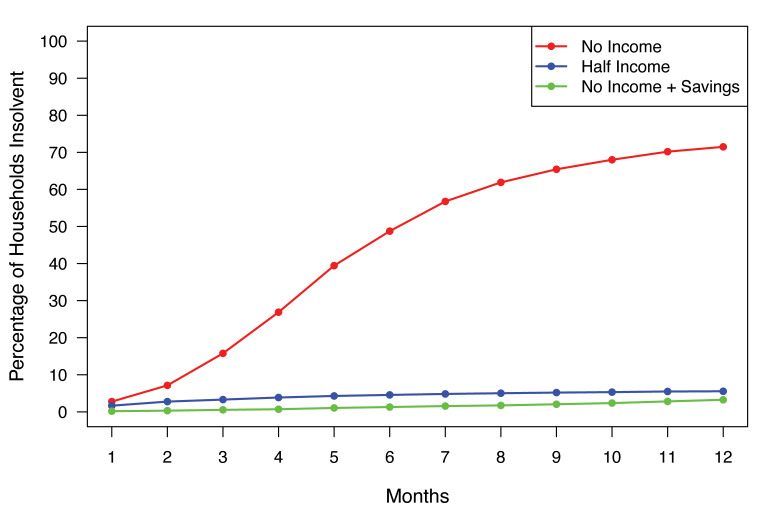
Household Insolvency Three income stress tests were performed. For Test 1 (red line), respondents had 3 months of complete income loss; Test 2 (blue line) 50% loss of income over 3 months; and Test 3 (green line) complete income loss for 3 months, but with the ability to liquidate retirement savings.

The demographics of the 2023 SHED respondents are summarized in **Table 2.** Of the total 11 400 respondents, 17% carried unpaid medical debt.^10^ A total of 5718 respondents were between 35 and 64 years old. **Figure 1** shows rates of insolvency for the three stress tests. In the first stress test, 16% of respondents were unable to pay their monthly expenses (ie, insolvent) after 3 months of complete income loss, and 49% were insolvent after 6 months with no income. In the second stress test a 50% reduction in income led to 3% of respondents being insolvent at 3 months, increasing to 5% at 6 months. In the third test, the ability to liquidate savings led to an insolvency rate of 0.5% at 3 months, increasing to 1.3% at 6 months. In the modeling of actuarially fair catastrophic income loss insurance premiums, the estimated aggregate premium for the population studied was $906 207 049 367 without social security disability income (SSDI) benefits, and $720 865 054 907 with an average $17 868 SSDI annual benefit.

**Table 2.** Demographic Characteristics of 2023 SHED Respondents

**Category**	**No.**	**%**
Sex		
Male	930	51.2
Female	2788	48.8
Age		
35-44 y	1863	32.6
45-54 y	1660	29.0
55-64 y	2195	38.4
Education		
Less than high school	303	5.3
High school grad or equivalent	1092	19.1
Certificate or technical degree	318	5.6
Some college	1539	26.9
Bachelor’s degree or higher	2466	43.1
Race/ethnicity		
White non-Hispanic	3711	64.9
Black non-Hispanic	656	11.5
Other non-Hispanic	312	5.5
2+ races, non-Hispanic	207	3.6
Hispanic	832	14.6

Abbreviation: SHED, Survey on Household Economics.

## DISCUSSION

Despite increases in rates of health insurance coverage, there remains significant risk of financial insolvency during cancer treatment. Modeling a 50% income loss to simulate benefits from existing public and/or private disability insurance programs led to a significantly smaller proportion of respondents falling into insolvency. To put the total premium of more than $900 billion (without supplementation from SSDI benefits) into perspective, the SSDI paid out $1.5 trillion in benefits in 2023, while receiving $1.2 trillion in revenue. The Social Security Administration Old-Age, Survivors, and Disability Insurance Trust Funds hold approximately $2.8 trillion in reserves, while paying out an average disability benefit of $17 868.^11,12^ The rational objections to this type of insurance product consider immediate household financial stressors that can overshadow the value of income security via payment of insurance premiums. Furthermore, other factors can result in a reduced or lost income unrelated to disability, such as being laid off. Mandating any sort of insurance program participation would face many political, ideological, and economic hurdles.

There are limitations in the interpretation of the study results. The annual SHED data provided by the Federal Reserve is most complete on the expense side with regards to housing costs, more so than in reporting student loan and medical expense data. Other core household expenses that are captured within consumer price indices are not reported within the SHED database. These expenses include essential items such as food, transportation, clothing, and education in part. It is important to recognize that even within these and more discretionary categories (eg, recreation, communications), significant changes in spending may occur when income is jeopardized and illness refocuses activities. A more longitudinal evolution of spending is not modeled in this study, and it is possible that real reduction in discretionary spending may add some protection against insolvency. While housing costs comprise a large proportion of household expenditures (33.3% of the consumption basket according to the Consumer Price Index calculation),^13^ insolvency rates within this study almost certainly reflect an underestimate of true insolvency rates. This study represents a “lower limit” of insolvency rates that can provide a valuable reference point. This is a first approximation of the “best-case scenario,” and as a nontrivial insolvency rate already deserves attention.

Moreover, the third stress test (50% reduction in income and ability to liquidate retirement savings) is an idealized scenario. In this test, savings data are extracted from the SHED variable by asking respondents, “What is the approximate total amount of your household’s savings and investments?” No further detail is available to understand how liquid such savings and investments may be. As SHED data reflect population level household finances, we can infer that a large proportion of savings and investments is either illiquid short term or simply inaccessible. For example, the Congressional Budget Office reported in 2022 approximately 21% of household wealth was in the form of defined contribution and defined benefits plans, and another 20% in accrued Social Security funds. Moreover, retirement holdings in the form of defined pension benefits may be entirely inaccessible, and investments held in defined contribution accounts often will incur substantial early withdrawal penalties that are indifferent to the reason for liquidation. Combined, this represents a large proportion of savings that would incur a high penalty or be inaccessible in an emergency cash flow crisis.^14^ For these reasons, the true liquidity and protective effect of retirement holdings is likely overestimated within this study. Additionally, we could not ascertain the amount of equity that survey respondents hold in their primary residences. For example, for the purposes of servicing debt, we could not estimate the ability to obtain a home equity line of credit. Housing is not commonly paid using credit (eg, with a consumer credit card). Thus, this consumption does require continuous liquidity. The true risk of insolvency is likely significantly higher, factoring in a broader basket of recurring expenses.

When modeling catastrophic income insurance, we assumed participants to be risk-averse, while real-world participants would likely exhibit a wide range of risk tolerance. In the context of disability or other casualty insurance products, it is intuitively more favorable for insurance payors to secure participation from a broad risk pool. Hence, the appeal of “universal” participation in a disability income insurance market. Certainly, this is an idealized situation, and lower participation rates may be disproportionately comprised of high-risk beneficiaries, which would raise premium rates that are already materially costly for many households.

Another limitation surrounds the fact that HNC affects patients of various socioeconomic demographics and comprises subtypes that carry different prognoses. The treatment of HNC may include surgery alone, surgery with radiotherapy, surgery with radiotherapy and systemic therapy, or systemic therapy with radiotherapy. Additional treatment protocols may include combinations of these three modalities.

The clinical programs of the investigators within this study largely serve patients with HNC. For this reason, we chose to generate financial welfare models using treatment time courses that are typical for this disease. Setting a known parameter for time of lost income was useful for realistic simulations. For example, a newly diagnosed HNC patient may undergo surgery, followed by postoperative recovery, and ideally begin adjuvant therapy (if indicated) within 6 weeks of surgery. Adjuvant therapy (eg, radiotherapy with or without systemic therapy) typically has an approximately 6-week duration. We used this 12-week treatment course duration as the base parameter for time of lost income, and expanded from there, because this is a common scenario in HNC. We certainly acknowledge that this is not the only treatment duration experienced by HNC patients and further note that some patients (a minority) may continue to work and generate some level of income during their treatment courses. We also assumed that all respondents had health insurance and that no individuals younger than 35 years of age were diagnosed with HNC. We intuited that a small proportion of persons under age 35 years comprise the 25% probability of disability among persons 20 to 64 years of age.

In summary, the economics of US health insurance continues to materially evolve. The premium tax credits that were a vital component of the Affordable Care Act are currently in jeopardy. Despite recent passage by the US House of Representatives of a bill to extend such credits for 3 more years, Senate approval awaits and is not a certainty. Without this credit, health insurance premiums will substantially rise for many households, another variable to factor into financial vulnerability analysis. This is not modeled in our analysis and would need to factor in both reduced household participation in health insurance markets, and greater monthly expenses, both of which would further increase the insolvency rate.

To focus our model on financial welfare exclusive of healthcare costs, we assumed health insurance coverage for all simulated cancer patients. This assumption is another reason that our model represents a base case upon which additional parameters such as health insurance status can be layered, as additional data become available. HNC can affect persons in a broad range of economic quantiles. Therefore, part of the value of modeling based SHED data, is to capture a population-level view of financial vulnerability. Future studies can also investigate financial vulnerability in more specific economic quantiles.

## CONCLUSION

The household economic impact of highly morbid diseases such as HNC highlight the need for more substantial income protection programs in parallel with health insurance coverage. Our model can be applied to other cancer types and conditions with varying expected durations of income loss. These preliminary data may help develop broader catastrophic income loss insurance products.

### Disclosures

The authors report no conflicts or funding.
